# 3-(4-Chloro­phen­yl)-1-cyclo­propyl-2-(2-fluoro­phen­yl)-5-phenyl­pentane-1,5-dione

**DOI:** 10.1107/S1600536813011276

**Published:** 2013-04-30

**Authors:** Thothadri Srinivasan, Govindaraj Senthilkumar, Haridoss Manikandan, Mannathusamy Gopalakrishnan, Devadasan Velmurugan

**Affiliations:** a Centre of Advanced Study in Crystallography and Biophysics, University of Madras, Guindy Campus, Chennai 600 025, India; b Department of Chemistry, Annamalai University, Annamalainagar 608 002, Tamilnadu, India

## Abstract

In the title compound, C_26_H_22_ClFO_2_, the cyclo­propane ring is disordered over two orientations, with site-occupancy factors of 0.64 (2) and 0.36 (2). The major occupancy component of the cyclo­propane ring makes dihedral angles of 47.6 (7), 50.4 (7) and 65.4 (7)° with the fluoro-, chloro- and unsubstituted benzene rings, respectively [the corresponding values for the minor occupancy component are 47.6 (12), 51.0 (12) and 60.9 (12)°]. An intra­molecular C—H⋯O hydrogen bond occurs. The F and Cl atoms deviate by 0.0508 (12) and 0.0592 (7) Å from the planes of their attached benzene rings. In the crystal, C—H⋯F hydrogen bonds link the mol­ecules into chains along the *b-*axis direction.

## Related literature

For the uses and biological importance of diketones, see: Bennett *et al.* (1999 ▶); Sato *et al.* (2008 ▶). For a related structure, see: Li *et al.* (2008 ▶).
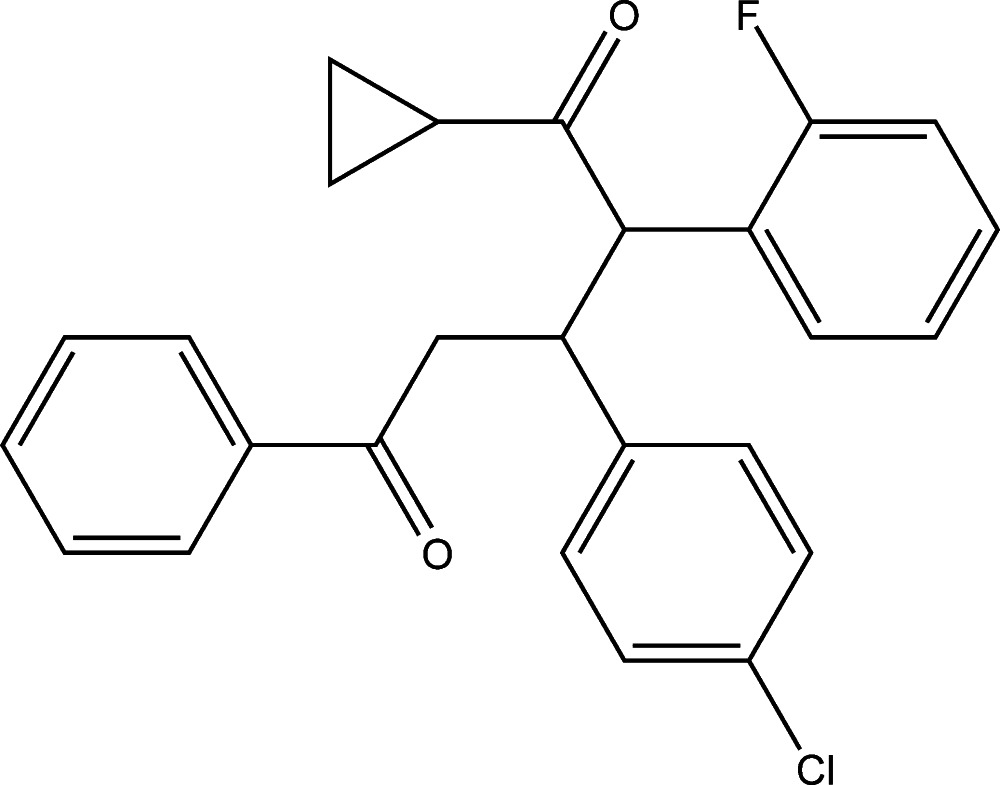


## Experimental

### 

#### Crystal data

C_26_H_22_ClFO_2_
*M* *_r_* = 420.89
Monoclinic, 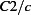

*a* = 40.0712 (18) Å
*b* = 5.6840 (2) Å
*c* = 18.6470 (8) Å
β = 92.903 (2)°
*V* = 4241.7 (3) Å^3^
*Z* = 8
Mo *K*α radiation
μ = 0.21 mm^−1^
*T* = 293 K
0.30 × 0.25 × 0.20 mm

#### Data collection

Bruker SMART APEXII area-detector diffractometer
Absorption correction: multi-scan (*SADABS*; Bruker, 2008 ▶) *T*_min_ = 0.940, *T*_max_ = 0.959
20059 measured reflections
5298 independent reflections
3397 reflections with *I* > 2σ(*I*)
*R*_int_ = 0.033

#### Refinement

*R*[*F* ^2^ > 2σ(*F* ^2^)] = 0.044
*wR*(*F* ^2^) = 0.125
*S* = 1.02
5298 reflections
299 parameters
40 restraints
H-atom parameters constrained
Δρ_max_ = 0.30 e Å^−3^
Δρ_min_ = −0.41 e Å^−3^

### 

Data collection: *APEX2* (Bruker, 2008 ▶); cell refinement: *SAINT* (Bruker, 2008 ▶); data reduction: *SAINT*; program(s) used to solve structure: *SHELXS97* (Sheldrick, 2008 ▶); program(s) used to refine structure: *SHELXL97* (Sheldrick, 2008 ▶); molecular graphics: *ORTEP-3 for Windows* (Farrugia, 2012) ▶; software used to prepare material for publication: *SHELXL97* and *PLATON* (Spek, 2009 ▶).

## Supplementary Material

Crystal structure: contains datablock(s) global, I. DOI: 10.1107/S1600536813011276/pv2628sup1.cif

Structure factors: contains datablock(s) I. DOI: 10.1107/S1600536813011276/pv2628Isup2.hkl

Supplementary material file. DOI: 10.1107/S1600536813011276/pv2628Isup3.cml

Additional supplementary materials:  crystallographic information; 3D view; checkCIF report

## Figures and Tables

**Table 1:** Hydrogen-bond geometry (Å, °)

*D*—H⋯*A*	*D*—H	H⋯*A*	*D*⋯*A*	*D*—H⋯*A*
C12—H12⋯F1^i^	0.98	2.52	3.440 (2)	155
C7—H7⋯O1	0.93	2.57	3.152 (2)	121

Symmetry code: (i) 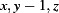.

**Fig. 1.**
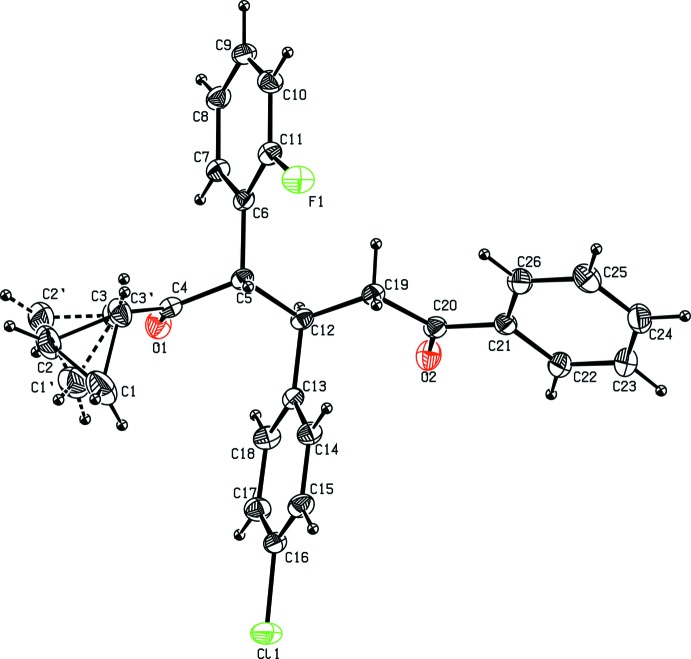
The molecular structure of the title compound, showing displacement ellipsoids drawn at the 30% probability level. H atoms are presented as small spheres of arbitrary radius. The minor components of the cyclopropane ring have been represented by broken bonds.

**Fig. 2.**
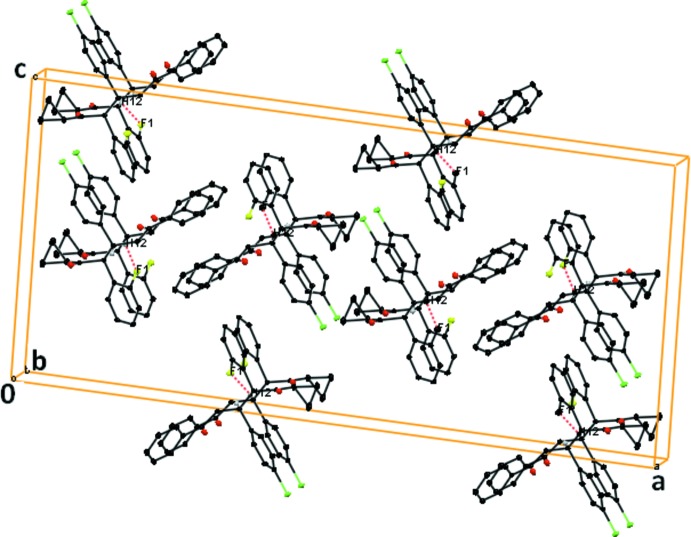
The crystal packing of the title compound viewed down b axis. H-atoms not involved in H-bonds have been excluded for clarity.

## supplementary crystallographic information

### Comment

Diketones are popular in organic synthesis for their applications in biology and medicine. They are known to exhibit antioxidants, antitumour and antibacterial activities (Bennett *et al.*, 1999). They are also key intermediates in the preparation of various heterocyclic compounds (Sato *et al.*, 2008).

In the title compound (Fig. 1), the cyclopropane ring (C1-C3) is disordered over two positions with the site occupancy factors of 0.64 (2):0.36 (2), representing to major and minor components, respectively. The cyclopropane ring (C1-C3) makes a dihedral angle of 47.6 (7)° with the fluoro substituted phenyl ring (C6-C11). It makes a dihedral angle of 50.4 (7)° with the chloro substituted phenyl ring (C13-C18) and a dihedral angle of 65.4 (7)° with the unsubstituted phenyl ring (C21-C26). The fluorine atom (F1) attached with the phenyl ring deviates by 0.0508 (12)Å.

The dihedral angle between the fluoro substituted phenyl ring and the chloro substituted phenyl ring is 6.50 (9)° and the dihedral angle between the fluoro substituted phenyl ring and the unsubstituted phenyl ring is 64.52 (10)°. The dihedral angle between the chloro substituted phenyl ring and the unsubstituted phenyl ring is 70.89 (10)°. The chlorine atom (Cl1) attached with the phenyl ring deviates by 0.0592 (7)Å. The packing of the crystal is stabilized by C–H···F hydrogen bonds.

### Experimental

A mixture of acetophenone (0.01 mole), 4-chlorobenzaldehyde (0.01 mole), cyclopropyl 2-fluorobenzyl ketone (0.01 mole) and sodium hydroxide solution (10 ml, 10%) in ethanol (50 ml) was stirred for 3 hrs at room temperature. The solid that separated was filtered and washed with distilled water. The product was recrystallised from ethanol. Yield=96%, melting point = 418-421 K. Single crystals suitable for X-ray diffraction were obtained by slow evaporation of a solution of the title compound in ethanol at room temperature.

### Refinement

The cyclopropane ring was disordered over two positions with the site occupancy factors of 0.64 (2):0.36 (2). The bond distances of the disordered components were restrained using standard similarity restraints SADI [SHELXL97, Sheldrick, 2008] with s.u. of 0.01 Å. The hydrogen atoms were placed in calculated positions with C—H = 0.93 to 0.98 Å and refined in the riding model with fixed isotropic displacement parameters: *U*_iso_(H) = 1.5*U*_eq_(C) for methyl group and *U*_iso_(H) = 1.2*U*_eq_(C) for other groups.

### Crystal data

**Table:**

C_26_H_22_ClFO_2_	*F*(000) = 1760
*M**_r_* = 420.89	*D*_x_ = 1.318 Mg m^−^^3^
Monoclinic, *C*2/*c*	Mo *K*α radiation, λ = 0.71073 Å
Hall symbol: -C 2yc	Cell parameters from 5298 reflections
*a* = 40.0712 (18) Å	θ = 2.0–28.4°
*b* = 5.6840 (2) Å	µ = 0.21 mm^−^^1^
*c* = 18.6470 (8) Å	*T* = 293 K
β = 92.903 (2)°	Block, colourless
*V* = 4241.7 (3) Å^3^	0.30 × 0.25 × 0.20 mm
*Z* = 8	

### Data collection

**Table:**

Bruker SMART APEXII area-detector diffractometer	5298 independent reflections
Radiation source: fine-focus sealed tube	3397 reflections with *I* > 2σ(*I*)
Graphite monochromator	*R*_int_ = 0.033
ω and φ scans	θ_max_ = 28.4°, θ_min_ = 2.0°
Absorption correction: multi-scan (*SADABS*; Bruker, 2008)	*h* = −53→46
*T*_min_ = 0.940, *T*_max_ = 0.959	*k* = −6→7
20059 measured reflections	*l* = −24→24

### Refinement

**Table:**

Refinement on *F*^2^	Primary atom site location: structure-invariant direct methods
Least-squares matrix: full	Secondary atom site location: difference Fourier map
*R*[*F*^2^ > 2σ(*F*^2^)] = 0.044	Hydrogen site location: inferred from neighbouring sites
*wR*(*F*^2^) = 0.125	H-atom parameters constrained
*S* = 1.02	*w* = 1/[σ^2^(*F*_o_^2^) + (0.0482*P*)^2^ + 2.4951*P*] where *P* = (*F*_o_^2^ + 2*F*_c_^2^)/3
5298 reflections	(Δ/σ)_max_ < 0.001
299 parameters	Δρ_max_ = 0.30 e Å^−^^3^
40 restraints	Δρ_min_ = −0.41 e Å^−^^3^

### Special details

**Table:**

Geometry. All esds (except the esd in the dihedral angle between two l.s. planes) are estimated using the full covariance matrix. The cell esds are taken into account individually in the estimation of esds in distances, angles and torsion angles; correlations between esds in cell parameters are only used when they are defined by crystal symmetry. An approximate (isotropic) treatment of cell esds is used for estimating esds involving l.s. planes.
Refinement. Refinement of F^2^ against ALL reflections. The weighted R-factor wR and goodness of fit S are based on F^2^, conventional R-factors R are based on F, with F set to zero for negative F^2^. The threshold expression of F^2^ > 2sigma(F^2^) is used only for calculating R-factors(gt) etc. and is not relevant to the choice of reflections for refinement. R-factors based on F^2^ are statistically about twice as large as those based on F, and R- factors based on ALL data will be even larger.

### Fractional atomic coordinates and isotropic or equivalent isotropic displacement parameters (Å^2^)

**Table:**

	*x*	*y*	*z*	*U*_iso_*/*U*_eq_	Occ. (<1)
C1	0.4715 (2)	0.5122 (16)	0.0640 (6)	0.096 (2)	0.64 (2)
H1A	0.4709	0.3917	0.0272	0.115*	0.64 (2)
H1B	0.4770	0.6687	0.0476	0.115*	0.64 (2)
C2	0.48333 (18)	0.4440 (17)	0.1374 (7)	0.087 (2)	0.64 (2)
H2A	0.4962	0.5588	0.1656	0.104*	0.64 (2)
H2B	0.4901	0.2820	0.1452	0.104*	0.64 (2)
C3	0.44683 (13)	0.4917 (11)	0.1227 (6)	0.061 (2)	0.64 (2)
H3	0.4378	0.6380	0.1417	0.074*	0.64 (2)
C1'	0.4818 (4)	0.455 (3)	0.0871 (9)	0.095 (3)	0.36 (2)
H1'1	0.4849	0.3187	0.0568	0.114*	0.36 (2)
H1'2	0.4923	0.5981	0.0711	0.114*	0.36 (2)
C2'	0.4808 (4)	0.416 (3)	0.1652 (9)	0.082 (3)	0.36 (2)
H2'1	0.4908	0.5347	0.1968	0.098*	0.36 (2)
H2'2	0.4834	0.2555	0.1826	0.098*	0.36 (2)
C3'	0.4489 (3)	0.477 (2)	0.1234 (11)	0.076 (4)	0.36 (2)
H3'	0.4405	0.6375	0.1296	0.092*	0.36 (2)
C4	0.42390 (4)	0.2929 (3)	0.11268 (8)	0.0460 (4)	
C5	0.38759 (4)	0.3590 (3)	0.09542 (8)	0.0401 (4)	
H5	0.3872	0.5188	0.0757	0.048*	
C6	0.36916 (4)	0.3639 (3)	0.16514 (8)	0.0402 (4)	
C7	0.37293 (5)	0.1868 (3)	0.21654 (8)	0.0471 (4)	
H7	0.3867	0.0594	0.2079	0.057*	
C8	0.35657 (5)	0.1965 (4)	0.28028 (9)	0.0538 (5)	
H8	0.3593	0.0755	0.3136	0.065*	
C9	0.33630 (5)	0.3840 (4)	0.29427 (9)	0.0548 (5)	
H9	0.3255	0.3907	0.3372	0.066*	
C10	0.33198 (5)	0.5618 (3)	0.24466 (10)	0.0539 (5)	
H10	0.3184	0.6900	0.2536	0.065*	
C11	0.34822 (5)	0.5464 (3)	0.18149 (9)	0.0460 (4)	
C12	0.37113 (4)	0.1936 (3)	0.03779 (8)	0.0412 (4)	
H12	0.3693	0.0370	0.0592	0.049*	
C13	0.39268 (4)	0.1719 (3)	−0.02688 (8)	0.0396 (4)	
C14	0.39404 (5)	0.3478 (3)	−0.07800 (9)	0.0483 (4)	
H14	0.3815	0.4839	−0.0730	0.058*	
C15	0.41382 (5)	0.3247 (4)	−0.13672 (9)	0.0547 (5)	
H15	0.4143	0.4434	−0.1710	0.066*	
C16	0.43258 (5)	0.1254 (4)	−0.14354 (9)	0.0512 (4)	
C17	0.43202 (5)	−0.0512 (4)	−0.09335 (10)	0.0568 (5)	
H17	0.4450	−0.1854	−0.0981	0.068*	
C18	0.41202 (5)	−0.0269 (3)	−0.03580 (10)	0.0524 (4)	
H18	0.4115	−0.1472	−0.0021	0.063*	
C19	0.33593 (4)	0.2781 (3)	0.01664 (9)	0.0471 (4)	
H19A	0.3234	0.2913	0.0596	0.056*	
H19B	0.3373	0.4340	−0.0042	0.056*	
C20	0.31696 (4)	0.1198 (3)	−0.03613 (9)	0.0462 (4)	
C21	0.28700 (4)	0.2161 (3)	−0.07669 (8)	0.0440 (4)	
C22	0.27314 (5)	0.0884 (4)	−0.13424 (10)	0.0583 (5)	
H22	0.2830	−0.0523	−0.1473	0.070*	
C23	0.24508 (5)	0.1663 (5)	−0.17219 (12)	0.0718 (6)	
H23	0.2364	0.0797	−0.2111	0.086*	
C24	0.22983 (5)	0.3707 (4)	−0.15316 (12)	0.0690 (6)	
H24	0.2104	0.4207	−0.1782	0.083*	
C25	0.24318 (5)	0.5017 (4)	−0.09711 (12)	0.0663 (6)	
H25	0.2330	0.6416	−0.0845	0.080*	
C26	0.27181 (5)	0.4263 (4)	−0.05916 (11)	0.0567 (5)	
H26	0.2809	0.5174	−0.0216	0.068*	
O1	0.43244 (4)	0.0882 (2)	0.11642 (7)	0.0627 (4)	
O2	0.32562 (4)	−0.0827 (2)	−0.04499 (8)	0.0632 (4)	
F1	0.34265 (3)	0.72072 (19)	0.13225 (6)	0.0685 (3)	
Cl1	0.456570 (16)	0.08923 (13)	−0.21778 (3)	0.0826 (2)	

### Atomic displacement parameters (Å^2^)

**Table:**

	*U*^11^	*U*^22^	*U*^33^	*U*^12^	*U*^13^	*U*^23^
C1	0.059 (3)	0.113 (4)	0.118 (4)	−0.011 (3)	0.019 (3)	0.030 (3)
C2	0.049 (3)	0.104 (4)	0.106 (5)	−0.015 (2)	−0.001 (4)	0.000 (5)
C3	0.044 (3)	0.055 (3)	0.084 (4)	−0.008 (3)	0.002 (3)	−0.013 (3)
C1'	0.069 (6)	0.128 (7)	0.091 (6)	−0.017 (5)	0.018 (5)	0.026 (6)
C2'	0.060 (5)	0.107 (6)	0.078 (6)	−0.021 (4)	0.000 (5)	0.006 (5)
C3'	0.062 (7)	0.094 (8)	0.074 (7)	−0.012 (6)	0.005 (6)	0.003 (6)
C4	0.0513 (10)	0.0538 (11)	0.0331 (7)	−0.0001 (9)	0.0035 (7)	−0.0024 (7)
C5	0.0479 (9)	0.0374 (8)	0.0352 (7)	−0.0046 (7)	0.0027 (6)	0.0009 (6)
C6	0.0447 (9)	0.0410 (9)	0.0345 (7)	−0.0061 (7)	−0.0004 (6)	−0.0028 (7)
C7	0.0522 (10)	0.0487 (10)	0.0404 (8)	0.0009 (8)	0.0019 (7)	0.0019 (7)
C8	0.0603 (11)	0.0629 (12)	0.0381 (8)	−0.0090 (10)	0.0010 (8)	0.0072 (8)
C9	0.0550 (11)	0.0699 (13)	0.0403 (9)	−0.0131 (10)	0.0094 (8)	−0.0109 (9)
C10	0.0535 (11)	0.0524 (11)	0.0566 (10)	−0.0019 (9)	0.0094 (8)	−0.0145 (9)
C11	0.0534 (10)	0.0403 (9)	0.0444 (8)	−0.0045 (8)	0.0015 (7)	−0.0017 (7)
C12	0.0506 (10)	0.0390 (8)	0.0343 (7)	−0.0064 (7)	0.0032 (6)	0.0005 (6)
C13	0.0449 (9)	0.0397 (9)	0.0341 (7)	−0.0055 (7)	0.0001 (6)	0.0002 (6)
C14	0.0578 (11)	0.0458 (10)	0.0415 (8)	0.0020 (8)	0.0034 (7)	0.0048 (7)
C15	0.0659 (12)	0.0577 (11)	0.0409 (9)	−0.0092 (10)	0.0057 (8)	0.0086 (8)
C16	0.0481 (10)	0.0661 (12)	0.0398 (8)	−0.0115 (9)	0.0062 (7)	−0.0094 (8)
C17	0.0609 (12)	0.0533 (11)	0.0567 (10)	0.0063 (9)	0.0078 (9)	−0.0075 (9)
C18	0.0680 (12)	0.0433 (10)	0.0464 (9)	0.0020 (9)	0.0073 (8)	0.0044 (8)
C19	0.0477 (10)	0.0527 (10)	0.0410 (8)	−0.0043 (8)	0.0044 (7)	−0.0070 (8)
C20	0.0484 (10)	0.0488 (10)	0.0417 (8)	−0.0093 (8)	0.0064 (7)	−0.0020 (8)
C21	0.0423 (9)	0.0481 (10)	0.0423 (8)	−0.0095 (8)	0.0079 (7)	0.0012 (7)
C22	0.0518 (11)	0.0647 (12)	0.0581 (11)	−0.0049 (9)	0.0001 (9)	−0.0122 (10)
C23	0.0571 (13)	0.0924 (17)	0.0646 (13)	−0.0050 (12)	−0.0094 (10)	−0.0107 (12)
C24	0.0518 (12)	0.0860 (16)	0.0686 (13)	−0.0023 (12)	−0.0044 (10)	0.0138 (12)
C25	0.0580 (13)	0.0586 (12)	0.0831 (14)	0.0038 (10)	0.0100 (11)	0.0095 (11)
C26	0.0561 (12)	0.0543 (11)	0.0599 (11)	−0.0073 (9)	0.0043 (9)	−0.0038 (9)
O1	0.0640 (9)	0.0567 (9)	0.0663 (8)	0.0095 (7)	−0.0058 (7)	−0.0002 (7)
O2	0.0662 (9)	0.0488 (8)	0.0732 (9)	−0.0011 (7)	−0.0107 (7)	−0.0074 (7)
F1	0.0872 (8)	0.0500 (6)	0.0693 (7)	0.0144 (6)	0.0138 (6)	0.0116 (6)
Cl1	0.0771 (4)	0.1152 (5)	0.0580 (3)	−0.0121 (3)	0.0268 (3)	−0.0149 (3)

### Geometric parameters (Å, º)

**Table:**

C1—C2	1.476 (7)	C10—H10	0.9300
C1—C3	1.515 (7)	C11—F1	1.3615 (19)
C1—H1A	0.9700	C12—C19	1.523 (2)
C1—H1B	0.9700	C12—C13	1.524 (2)
C2—C3	1.499 (5)	C12—H12	0.9800
C2—H2A	0.9700	C13—C18	1.385 (2)
C2—H2B	0.9700	C13—C14	1.384 (2)
C3—C4	1.463 (4)	C14—C15	1.390 (2)
C3—H3	0.9800	C14—H14	0.9300
C1'—C2'	1.475 (9)	C15—C16	1.369 (3)
C1'—C3'	1.516 (8)	C15—H15	0.9300
C1'—H1'1	0.9700	C16—C17	1.373 (3)
C1'—H1'2	0.9700	C16—Cl1	1.7371 (17)
C2'—C3'	1.505 (9)	C17—C18	1.379 (3)
C2'—H2'1	0.9700	C17—H17	0.9300
C2'—H2'2	0.9700	C18—H18	0.9300
C3'—C4	1.458 (7)	C19—C20	1.510 (2)
C3'—H3'	0.9800	C19—H19A	0.9700
C4—O1	1.214 (2)	C19—H19B	0.9700
C4—C5	1.521 (2)	C20—O2	1.216 (2)
C5—C6	1.528 (2)	C20—C21	1.491 (2)
C5—C12	1.550 (2)	C21—C26	1.387 (3)
C5—H5	0.9800	C21—C22	1.388 (2)
C6—C11	1.378 (2)	C22—C23	1.372 (3)
C6—C7	1.393 (2)	C22—H22	0.9300
C7—C8	1.387 (2)	C23—C24	1.368 (3)
C7—H7	0.9300	C23—H23	0.9300
C8—C9	1.373 (3)	C24—C25	1.370 (3)
C8—H8	0.9300	C24—H24	0.9300
C9—C10	1.375 (3)	C25—C26	1.385 (3)
C9—H9	0.9300	C25—H25	0.9300
C10—C11	1.377 (2)	C26—H26	0.9300
			
C2—C1—C3	60.1 (3)	C10—C9—H9	120.1
C2—C1—H1A	117.8	C9—C10—C11	118.67 (17)
C3—C1—H1A	117.8	C9—C10—H10	120.7
C2—C1—H1B	117.8	C11—C10—H10	120.7
C3—C1—H1B	117.8	F1—C11—C10	117.53 (16)
H1A—C1—H1B	114.9	F1—C11—C6	118.65 (14)
C1—C2—C3	61.2 (3)	C10—C11—C6	123.81 (16)
C1—C2—H2A	117.6	C19—C12—C13	112.05 (13)
C3—C2—H2A	117.6	C19—C12—C5	110.16 (13)
C1—C2—H2B	117.6	C13—C12—C5	111.07 (13)
C3—C2—H2B	117.6	C19—C12—H12	107.8
H2A—C2—H2B	114.8	C13—C12—H12	107.8
C4—C3—C2	119.0 (6)	C5—C12—H12	107.8
C4—C3—C1	113.3 (6)	C18—C13—C14	117.70 (15)
C2—C3—C1	58.6 (3)	C18—C13—C12	120.29 (14)
C4—C3—H3	117.5	C14—C13—C12	122.00 (15)
C2—C3—H3	117.5	C13—C14—C15	121.21 (17)
C1—C3—H3	117.5	C13—C14—H14	119.4
C2'—C1'—C3'	60.4 (4)	C15—C14—H14	119.4
C2'—C1'—H1'1	117.7	C16—C15—C14	119.26 (17)
C3'—C1'—H1'1	117.7	C16—C15—H15	120.4
C2'—C1'—H1'2	117.7	C14—C15—H15	120.4
C3'—C1'—H1'2	117.7	C15—C16—C17	120.91 (16)
H1'1—C1'—H1'2	114.9	C15—C16—Cl1	120.03 (15)
C1'—C2'—C3'	61.2 (4)	C17—C16—Cl1	119.04 (16)
C1'—C2'—H2'1	117.7	C16—C17—C18	119.19 (18)
C3'—C2'—H2'1	117.7	C16—C17—H17	120.4
C1'—C2'—H2'2	117.7	C18—C17—H17	120.4
C3'—C2'—H2'2	117.7	C17—C18—C13	121.73 (17)
H2'1—C2'—H2'2	114.8	C17—C18—H18	119.1
C4—C3'—C2'	117.6 (13)	C13—C18—H18	119.1
C4—C3'—C1'	119.1 (10)	C20—C19—C12	114.19 (15)
C2'—C3'—C1'	58.4 (4)	C20—C19—H19A	108.7
C4—C3'—H3'	116.4	C12—C19—H19A	108.7
C2'—C3'—H3'	116.4	C20—C19—H19B	108.7
C1'—C3'—H3'	116.4	C12—C19—H19B	108.7
O1—C4—C3'	119.4 (7)	H19A—C19—H19B	107.6
O1—C4—C3	124.0 (3)	O2—C20—C21	120.46 (16)
O1—C4—C5	120.85 (16)	O2—C20—C19	121.01 (17)
C3'—C4—C5	119.7 (7)	C21—C20—C19	118.53 (15)
C3—C4—C5	115.1 (3)	C26—C21—C22	118.03 (17)
C4—C5—C6	108.84 (12)	C26—C21—C20	123.26 (16)
C4—C5—C12	111.47 (14)	C22—C21—C20	118.70 (16)
C6—C5—C12	113.19 (13)	C23—C22—C21	121.0 (2)
C4—C5—H5	107.7	C23—C22—H22	119.5
C6—C5—H5	107.7	C21—C22—H22	119.5
C12—C5—H5	107.7	C24—C23—C22	120.4 (2)
C11—C6—C7	115.96 (15)	C24—C23—H23	119.8
C11—C6—C5	121.87 (14)	C22—C23—H23	119.8
C7—C6—C5	122.16 (15)	C23—C24—C25	119.8 (2)
C8—C7—C6	121.44 (17)	C23—C24—H24	120.1
C8—C7—H7	119.3	C25—C24—H24	120.1
C6—C7—H7	119.3	C24—C25—C26	120.2 (2)
C9—C8—C7	120.20 (17)	C24—C25—H25	119.9
C9—C8—H8	119.9	C26—C25—H25	119.9
C7—C8—H8	119.9	C25—C26—C21	120.50 (19)
C8—C9—C10	119.89 (16)	C25—C26—H26	119.7
C8—C9—H9	120.1	C21—C26—H26	119.7
			
C1—C2—C3—C4	101.0 (8)	C5—C6—C11—C10	−177.77 (16)
C2—C1—C3—C4	−110.8 (7)	C4—C5—C12—C19	−174.70 (13)
C1'—C2'—C3'—C4	108.8 (13)	C6—C5—C12—C19	62.20 (17)
C2'—C1'—C3'—C4	−106.3 (17)	C4—C5—C12—C13	−49.96 (18)
C2'—C3'—C4—O1	−23 (2)	C6—C5—C12—C13	−173.05 (13)
C1'—C3'—C4—O1	44 (2)	C19—C12—C13—C18	−133.75 (17)
C2'—C3'—C4—C3	155 (17)	C5—C12—C13—C18	102.58 (18)
C1'—C3'—C4—C3	−138 (17)	C19—C12—C13—C14	46.9 (2)
C2'—C3'—C4—C5	158.4 (12)	C5—C12—C13—C14	−76.75 (19)
C1'—C3'—C4—C5	−134.2 (14)	C18—C13—C14—C15	0.7 (3)
C2—C3—C4—O1	1.1 (11)	C12—C13—C14—C15	−179.92 (16)
C1—C3—C4—O1	66.9 (9)	C13—C14—C15—C16	−0.8 (3)
C2—C3—C4—C3'	−1 (15)	C14—C15—C16—C17	0.1 (3)
C1—C3—C4—C3'	64 (15)	C14—C15—C16—Cl1	178.20 (14)
C2—C3—C4—C5	−177.9 (7)	C15—C16—C17—C18	0.6 (3)
C1—C3—C4—C5	−112.0 (7)	Cl1—C16—C17—C18	−177.55 (15)
O1—C4—C5—C6	86.48 (19)	C16—C17—C18—C13	−0.6 (3)
C3'—C4—C5—C6	−94.9 (9)	C14—C13—C18—C17	−0.1 (3)
C3—C4—C5—C6	−94.5 (5)	C12—C13—C18—C17	−179.41 (17)
O1—C4—C5—C12	−39.1 (2)	C13—C12—C19—C20	59.33 (19)
C3'—C4—C5—C12	139.6 (9)	C5—C12—C19—C20	−176.48 (13)
C3—C4—C5—C12	139.9 (5)	C12—C19—C20—O2	18.5 (2)
C4—C5—C6—C11	133.68 (16)	C12—C19—C20—C21	−162.26 (14)
C12—C5—C6—C11	−101.78 (18)	O2—C20—C21—C26	166.47 (17)
C4—C5—C6—C7	−45.1 (2)	C19—C20—C21—C26	−12.7 (2)
C12—C5—C6—C7	79.41 (19)	O2—C20—C21—C22	−12.5 (3)
C11—C6—C7—C8	−0.3 (2)	C19—C20—C21—C22	168.31 (16)
C5—C6—C7—C8	178.58 (15)	C26—C21—C22—C23	−0.6 (3)
C6—C7—C8—C9	−0.5 (3)	C20—C21—C22—C23	178.36 (19)
C7—C8—C9—C10	0.6 (3)	C21—C22—C23—C24	−1.1 (3)
C8—C9—C10—C11	0.2 (3)	C22—C23—C24—C25	1.8 (3)
C9—C10—C11—F1	177.85 (16)	C23—C24—C25—C26	−0.8 (3)
C9—C10—C11—C6	−1.1 (3)	C24—C25—C26—C21	−1.0 (3)
C7—C6—C11—F1	−177.82 (15)	C22—C21—C26—C25	1.7 (3)
C5—C6—C11—F1	3.3 (2)	C20—C21—C26—C25	−177.30 (17)
C7—C6—C11—C10	1.1 (3)		

### Hydrogen-bond geometry (Å, º)

**Table:**

*D*—H···*A*	*D*—H	H···*A*	*D*···*A*	*D*—H···*A*
C12—H12···F1^i^	0.98	2.52	3.440 (2)	155
C5—H5···F1	0.98	2.41	2.840 (2)	106
C7—H7···O1	0.93	2.57	3.152 (2)	121

Symmetry code: (i) *x*, *y*−1, *z*.

## References

[bb1] Bennett, I., Broom, N. J. P., Cassels, R., Elder, J. S., Masson, N. D. & O’Hanlon, P. J. (1999). *Bioorg. Med. Chem. Lett.* **9**, 1847–1852. 10.1016/s0960-894x(99)00296-6 10406653

[bb2] Bruker (2008). *APEX2*, *SAINT* and *SADABS* Bruker AXS Inc., Madison, Wisconsin, USA.

[bb3] Farrugia, L. J. (2012). *J. Appl. Cryst.* **45**, 849–854.

[bb4] Li, K.-Z., Chen, Y.-T., Zhao, C.-W., Wei, G.-D. & He, Q.-P. (2008). *Acta Cryst.* E**64**, o1665. 10.1107/S1600536808023970 PMC2960541 21201658

[bb5] Sato, K., Yamazoe, S., Yamamoto, R., Ohata, S., Tarui, A., Omote, M., Kumadaki, I. & Ando, A. (2008). *Org. Lett.* **10**, 2405–2408. 10.1021/ol800660y 18476711

[bb6] Sheldrick, G. M. (2008). *Acta Cryst.* A**64**, 112–122. 10.1107/S0108767307043930 18156677

[bb7] Spek, A. L. (2009). *Acta Cryst.* D**65**, 148–155. 10.1107/S090744490804362X PMC2631630 19171970

